# Sport and physical activity following unicompartmental knee arthroplasty: a systematic review

**DOI:** 10.1007/s00167-016-4167-1

**Published:** 2016-05-21

**Authors:** Wenzel Waldstein, Paul Kolbitsch, Ulrich Koller, Friedrich Boettner, Reinhard Windhager

**Affiliations:** 1 Department of Orthopaedics, Vienna General Hospital, Medical University of Vienna, Waehringer Guertel 18-20, 1090 Vienna, Austria; 2 Adult Reconstruction and Joint Replacement Division, Hospital for Special Surgery, New York, NY USA

**Keywords:** Unicompartmental knee arthroplasty, Sport, Physical activity, Review

## Abstract

**Purpose:**

Unicompartmental knee arthroplasty (UKA) can be a surgical treatment option for patients with high expectations regarding the post-operative level of physical activity. A systematic review was undertaken to answer three research questions: (1) is there an improvement of physical activity based on validated activity scores following UKA? (2) What are the sport disciplines and the sport patterns of UKA patients? (3) What are the pre- and post-operative sport participation rates and the return to activity rates of UKA patients?

**Methods:**

Following the PRISMA guidelines, EMBASE, MEDLINE, ISI Web of Science and the Cochrane Central Register of Controlled Trials were searched for studies reporting the level of sport and/or physical activity before and after UKA, and/or included at least one activity score before and after UKA.

**Results:**

Seventeen studies were identified reporting on 2972 UKAs, of which 89 % were medial UKAs and 92 % were mobile-bearing implants, respectively. Ten studies reported a statistically significant improvement of physical activity following UKA according to the UCLA activity score, the Tegner activity score or the High Activity Arthroplasty Score, respectively. Hiking, cycling and swimming are the most common activities following UKA. Sport participation before the onset of restricting symptoms ranged from 64 to 93 % and slightly decreased by 2–9 % following UKA. The return to activity rate ranged from 87 to 98 %.

**Conclusion:**

Patients following UKA are physically active according to validated activity scores. A significant increase in low-impact activities and a decrease in high-impact activities after UKA was observed. Patients with a UKA regularly participate in sports; however, sport participation slightly decreased compared to pre-arthritic levels. This systematic review helps physicians to manage the expectations of patients regarding the level of physical activity following UKA.

**Level of evidence:**

III.

## Introduction

Knee osteoarthritis (OA) is a frequent cause of pain and disability in the elderly [6, 9, 39]. Epidemiologic studies suggest that almost half of the general US population may develop symptomatic knee OA by the age of 85 years, with a particularly high risk in the obese [23]. The demand for primary knee arthroplasties is, therefore, projected to grow by more than 600 % by 2030 [20].

Unicompartmental knee arthroplasty (UKA) is an effective and safe treatment option for isolated end-stage medial or lateral compartment knee OA [5, 28]. Kozinn and Scott [19] proposed indications and contraindications for fixed-bearing UKAs. The authors postulated that damage to the patellofemoral joint, obesity, youth and high activity levels should be considered a contraindication for a UKA. However, for mobile-bearing implants most of these contraindications may be neglected. The Oxford group [24] proposed to consider an Oxford UKA in all knees with bone-on-bone arthritis [11], full-thickness cartilage in the contralateral compartment [11], a fully correctible intra-articular deformity [42] and an intact anterior cruciate ligament [43, 48]. Patellofemoral arthritis is no contraindication unless deep eburnation, bone loss or patellar subluxation is observed [24]. These criteria are satisfied in a significant proportion of knees requiring arthroplasty. For fixed-bearing UKAs, the indications are less defined. Deschamps and Chol [7] suggested that for modern fixed-bearing implants, indications are slightly stricter than for mobile-bearing implants with anterior knee pain and a body mass index above 30 kg/m^2^ being a (relative) contraindication.

For both UKA design concepts, excellent clinical results and improved survivorship have been reported [16, 27, 31, 40, 41, 46]. Consequently, UKAs are increasingly being used in younger and more active patients [2, 3]. This particular patient cohort, however, has high expectations concerning the post-operative level of physical activity [4, 8].

Conventional outcome scores such as the American Knee Society Score [17] assess objective parameters and what a patient is capable of doing. However, these rating scales do not take into account what a patient is actually doing [51]. In times of high post-operative expectations, activity rating scales are becoming a key factor to assess the outcome of a procedure. There has been no review analysing all available studies with regard to pre- and post-operative activity scores following UKA. Therefore, the present study undertook a systematic review analysing the level of sport and physical activity following UKA. The following research questions were asked: (1) is there an improvement of physical activity based on validated activity scores following UKA? (2) What are the sport disciplines and the sport patterns of UKA patients? (3) What are the pre- and post-operative sport participation rates and the return to activity rates of UKA patients?

## Materials and methods

EMBASE, MEDLINE and the ISI Web of Science for randomized controlled trials, quasi-randomized trials and controlled clinical trials as well as case series investigating the clinical outcome of UKAs with regard to physical activity and sport were searched. The inclusion criteria were defined as follows: studies reporting on the level of physical activity or sport before and after UKA using an activity score or an activity questionnaire. A combination of Medical Subject Headings (MESH) terms and free text was used to search for relevant studies. Studies in English or German language were identified (“Appendix”).

Two authors (P.K. and W.W.) reviewed all abstracts. The full text was analysed for studies meeting the inclusion criteria. The systematic review was conducted according to the preferred reporting items for systematic reviews and meta-analyses (PRISMA) guidelines [22]. The methodological index for non-randomized studies (MINORS) was used to assess the quality of all studies [34] (Table 1).

**Table 1:** Patient demographics and studies details extracted from studies included in the review (*n* = 17)

Patient demographics and study details											
References	Journal	No. of patients (knees)	Gender (per cent males)	Mean age (years)	Study type (prospective/retrospective)	Mean follow-up (months)	MINORS	Medial/lateral UKA	Type of bearing	Type of prosthesis	Type of fixation
Schai et al. [33]	JOA	28 (28)	39.2	52 (range 37–60)	Retrospective	40 (range 24–72)	9/16	*n* = 25 medial, *n* = 3 lateral	Fixed	PFC unicompartmental knee system (Johnson & Johnson, Raynham, MA, USA)	Cemented
Fisher et al. [10]	The Knee	66 (71)	48.5	64 (range 49–81)	Prospective	18 (range 4–46)	11/16	Medial	Mobile	Oxford phase 3 unicompartmental knee prosthesis (Zimmer Biomet, Bridgend, UK)	Cemented
Walton et al. [46]	J Knee Surg.	150 (183)	50.7	71.5 (range 36–92)	Retrospective	Minimum 12	11/24	Medial	Mobile	Oxford phase 3 unicompartmental knee prosthesis (Zimmer Biomet, Bridgend, UK)	cemented
Naal et al. [25]	AMJSM	83 (83)	54.2	65.5 (range 47–83)	Retrospective	18 (range 12–28)	10/16	*n* = 77 medial *n* = 6 lateral	Fixed	Preservation prosthesis (DePuy International Ltd, Leeds, UK)	Tibia cemented; femoral component not specified
Hopper and Leach [16]	KSSTA	34 (41)	58.8	61.3 (range 43–75)	Retrospective	22.3 (range 12–44)	13/24	Not specified	not specified	Oxford (Zimmer Biomet, Bridgend, UK)	Not specified
Naal et al. [26]	AOTS	77 (83)	55.8	66 (range 46–84)	Retrospective	Minimum 24	10/16	Medial	Fixed	Preservation prosthesis (DePuy International Ltd, Leeds, UK)	Not specified
Pandit et al. [28, 29]	BJJ/JBJS Br	818 (1000)	48	66 (range 32–88)	Prospective	Minimum 12	11/16	Medial	Mobile	Oxford phase 3 unicompartmental knee prosthesis (Zimmer Biomet, Bridgend, UK)	Cemented
Yim et al. [50]	JOA	50 (50)	4	60.3 (range 47–65)	Retrospective	44 (rage 36–48)	20/24	Medial	Fixed	Miller-Galante (Zimmer, Warsaw, IN, USA)	Cemented
Liddle et al. [21]	KSSTA	380 (406)	55	64.8 (range 35–87)	Prospective	Minimum 12	14/16	Medial	Mobile	Oxford phase 3 unicompartmental knee prosthesis (Zimmer Biomet, Bridgend, UK)	Not specified
Pandit et al. [30]	JBJS	62 (63)	63: cemented 53: cementless	63.8 (range 46–78) Cemented 64.7 (45–82) Cementless	Prospective	Minimum 60	23/24	Medial	Mobile	Oxford phase 3 Unicompartmental knee prosthesis (Zimmer Biomet, Bridgend, UK)	*n* = 33 cemented *n* = 30 cementless
Pietschmann et al. [32]	Int. Orthop.	171 (181)	44	65.3 (range 44–90)	Retrospective	50.4 (range 12–120)	7/16	Medial	Mobile	Oxford phase 3 unicompartmental knee prosthesis (Zimmer Biomet, Bridgend, UK)	Cemented
Jahnke et al. [18]	Int. Orthop.	159 (178)	52.8	63.5 (range 36–86)	Prospective	24 (range 1.47)	8/16	Medial	Mobile	Oxford (Zimmer Biomet, Bridgend, UK)	Not specified
Weston-Simons et al. [47]	BJJ	258 (265)	35.2	63.6 (range 32–90)	Prospective	49.2 (range 6–99.6)	14/16	Lateral	Mobile	Oxford phase 3 domed unicompartmental knee prothesis (Zimmer Biomet, Bridgend, UK)	Cemented
Walker et al. [44]	KSSTA	45 (45)	42.2	60.1 (range 36–81)	Retrospective	35.4 (range 24–51)	11/16	Lateral	Mobile	Oxford (Zimmer Biomet, Bridgend, UK)	Cemented
Walker et al. [45]	JOA	93 (109)	49.4	55 (range 26–60)	Prospective	52.8 (range 27.6–100.8)	10/16	Medial	Mobile	Oxford phase 3 unicompartmental knee prosthesis (Zimmer Biomet, Bridgend, UK)	Cemented
Hooper et al. [15]	BJJ	126 (150)	53.1	63.6 (range 39–86)	Prospective	Minimum 60	11/16	Medial	Mobile	Oxford phase 3 unicompartmental knee prosthesis (Zimmer Biomet, Bridgend, UK)	Uncemented
Ho et al. [14]	J Knee Surg.	36 (36)	33	60.0 (range 53–64)	Retrospective	45.6 (range 24–68.4)	15/24	Medial	Not specified	Not specified	Not specified

The methodological quality of each study was evaluated using the methodological index for non-randomized studies (MINORS)

*JOA* Journal of Arthroplasty; *AMJSM* American Journal of Sports Medicine; *KSSTA* Knee Surgery, Sports Traumatology, Arthroscopy; *AOTS* Archives of Orthopaedic and Trauma Surgery; *BJJ* Bone and Joint Journal; *JBJS* Journal of Bone and Joint Surgery; *J Knee Surg.* Journal of Knee Surgery

Data were extracted using a standardized form, including patient demographics follow-up period, type of implant, and scores or questionnaires assessing pre- and post-operative activity levels. The same activity score had to be performed pre-operatively and at least one time post-operatively. There were no restrictions regarding type of score used. Duplicate articles and studies not reporting on the same pre- and post-operative scores were excluded. Additional information regarding physical activity or sport before and after UKA was documented, and used for further interpretation of the results.

### Statistical analysis

Descriptive statistics for the included studies were presented.

## Results

**Fig. 1:**
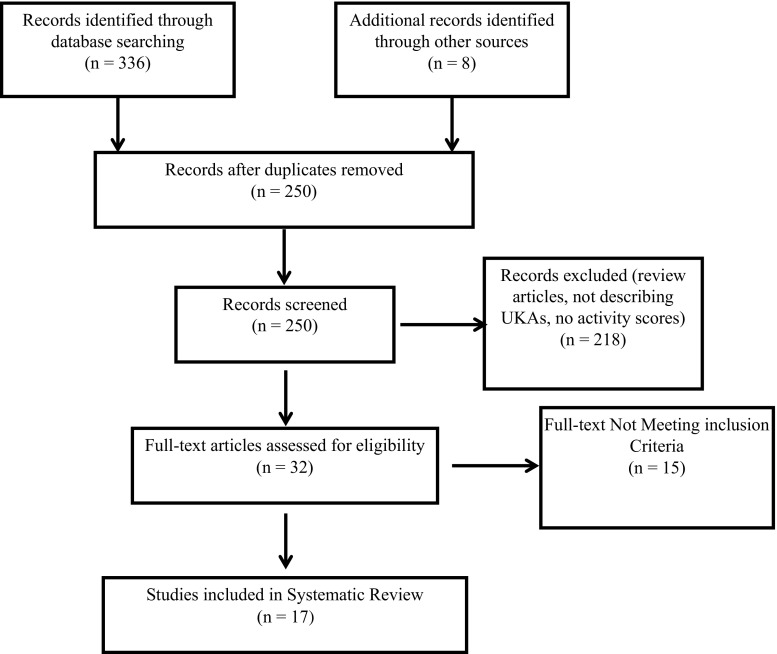
Flow chart of study protocol following the PRISMA guidelines. The systematic review included 17 studies

Three hundred and thirty-six studies were identified in the search. Additional eight studies were identified through cross-referencing. After exclusion of duplicates, a total of 250 papers were screened for applicability (Fig. 1). One study by Streit et al. [37] in 2012 reported on the same cohort as Walker et al. [44] in 2015; the study of Streit et al. [37] was therefore excluded from this review. Another study by Streit et al. [36] was not considered as it analysed the same cohort as Walker et al. [45]. Ultimately, 17 studies were included in the final analysis. These 17 studies reported on 2972 UKAs in 2636 patients. The patients’ age ranged from 25 to 92 years (Table 1). The majority of cases (89 %, 2653 of 2972) were medial UKAs; 11 % (319 of 2972) were lateral UKAs. One study analysing 41 UKAs did not specify whether a medial or lateral UKA was performed [16]. The mean MINORS score was of 11/16 points for single-arm studies (*n* = 12) and 16/24 points for comparative studies (*n* = 5) (Table 2).

**Table 2:** Methodological index for non-randomized studies (MINORS)

References	1	2	3	4	5	6	7	8	9	10	11	12	TOT
Schai et al. [33]	2	0	1	2	0	2	2	0	–	–	–	–	9/16
Fisher et al. [10]	2	2	2	2	0	2	1	0	–	–	–	–	11/16
Walton et al. [46]	0	0	1	1	0	2	1	0	2	1	2	1	11/24
Naal et al. [25]	2	2	1	2	0	2	1	0	–	–	–	–	10/16
Hopper and Leach [16]	2	2	1	1	0	1	1	0	1	2	1	1	13/24
Naal et al. [26]	2	2	1	2	0	1	2	0	–	–	–	–	10/16
Pandit et al. [28, 29]	2	2	2	2	2	1	0	0	–	–	–	–	11/16
Yim et al. [50]	2	1	2	2	0	2	2	2	1	2	2	2	20/24
Liddle et al. [21]	2	2	2	2	2	2	2	0	–	–	–	–	14/16
Pandit et al. [30]	2	0	2	2	2	2	2	2	2	2	2	2	23/24
Pietschmann et al. [32]	1	2	1	1	0	2	0	0	–	–	–	–	7/16
Jahnke et al. [18]	2	0	2	2	0	1	1	0	–	–	–	–	8/16
Weston-Simons et al. [47]	2	2	2	2	2	2	2	0	–	–	–	–	14/16
Walker et al. [44]	2	2	1	2	0	2	2	0	–	–	–	–	11/16
Walker et al. [45]	2	2	1	2	0	2	1	0	–	–	–	–	10/16
Hooper et al. [15]	2	2	2	2	0	2	1	0	–	–	–	–	11/16
Ho et al. [14]	2	0	1	2	0	2	1	0	1	2	2	2	15/24

The methodological index for non-randomized studies (MINORS) applied to all studies assessing the level physical activity before and after implantation of a unicompartmental knee arthroplasty. The items are scored 0 (not reported), 1 (reported but inadequate) or 2 (reported and adequate)

### Activity scores

Thirteen of seventeen studies used validated activity scores to evaluate the pre- and post-operative level of physical activity. Ten of these studies reported a statistically significant improvement following UKA according to the UCLA activity score [51], the Tegner activity score [38] or the High Activity Arthroplasty Score [1, 10, 15, 18, 21, 26, 30, 44, 45, 47] (Table 3).

**Table 3:** Overview of pre- and post-operative activity scores and sport questionnaires of all studies analysed

Activity scores and sport questionnaires						
References	Journal	Outcome score(s)	Pre-operative assessment (time)	Pre-operative values	Post-operative values	Significance
Schai et al. [33]	JOA	Tegner	Before surgery	2.3	2.7	Not calculated
Fisher et al. [10]	The Knee	UCLA	Before surgery	4.2 (range 3–6)	6.5 (range 3–9)	*p* < 0.01
Walton et al. [46]	J Knee Surg.	Simple sport questionnaire (no. of disciplines)	Before surgery	N/A	N/A	Not calculated
Naal et al. [25]	AMJSM	Sport questionnaire (no. of disciplines/frequency/session length)	Before onset of restricting symptoms	5/2.9/66 min	3.1/2.8/55 min	*p* < 0.01/0.329/0.08
Hopper and Leach [16]	KSSTA	Sport questionnaire (no. of disciplines/frequency per week/minimum session length)	Before surgery	1.5/3.2/85 min	1.4/3.4/92.1 min	*p* = 0.083/*p* = 0.727/*p* = 0.487
Naal et al. [26]	AOTS	UCLA	Before surgery	4.7 ± 2.4	7.1 ± 1.9	*p* < 0.01
Pandit et al. [28, 29]	BJJ/JBJS Br	Tegner	Before surgery	2.3 ± 1.1	1 year follow-up: 2.9 ± 0.9 10 years follow-up: 2 ± 0.8	Not calculated
Yim et al. [50]	JOA	Tegner, sport questionnaire (no. of disciplines)	Before surgery	3.2 ± 0.9 2.2 ± 1.6	2.6 ± 0.9 1.6 ± 1.7	Not calculated
Liddle et al. [21]	KSSTA	Tegner	Before surgery	2.0 (1–5)	3.0 (1–6)	*p* < 0.01
Pandit et al. [30]	JBJS	Tegner	Before surgery	Cemented: 1.9 ± 0.8 Cementless: 1.9 ± 0.7	2.6 ± 0.8 2.9 ± 0.6	*p* < 0.05 *p* < 0.05
Pietschmann et al. [32]	Int. Orthop.	Sport questionnaire (no. of disciplines/frequency per week/minimum session length)	Before surgery	1.73/–/–	1.74/–/–	Not calculated
Jahnke et al. [18]	Int. Orthop.	UCLA, Tegner, Heidelberg Sports Activity Score	Before surgery	6.2 ± 1.3 4.1 ± 1.4 23.8 ± 18.5	6.3 ± 1.1 4.0 ± 1.0 29.3 ± 18.5	*p* < 0.01 *p* = 0.27 *p* < 0.01
Weston-Simons et al. [17]	BJJ	Tegner	Before surgery	2.2 ± 1.2	2.9 ± 1.2	*p* < 0.01
Walker et al. [44]	KSSTA	UCLA, Tegner, Schulthess Clinic Activity Score	Before surgery Before surgery Before onset of restricting symptoms	5.3 ± 2.3 2.9 ± 1.6 N/A	6.7 ± 1.5 3.5 ± 0.8 N/A	*p* < 0.01 *p* < 0.01 N/A
Walker et al. [45]	JOA	UCLA, Tegner, Schulthess Clinic Activity Score	Before surgery Before surgery Before onset of restricting symptoms	3.3 ± 1.5 2.0 ± 1.1 N/A	6.8 ± 1.5 3.8 ± 1.1 N/A	*p* < 0.001 *p* < 0.001 N/A
Hooper et al. [15]	BJJ	High Activity Arthroplasty Score	Before surgery	4.33 ± 2.5	10.55 ± 1.93	*p* < 0.001
Ho et al. [14]	J Knee Surg.	UCLA	Before onset of restricting symptoms	8.1 ± 1.5	7.4 ± 1.6	Not calculated

*JOA* Journal of Arthroplasty; *AMJSM* American Journal of Sports Medicine; *KSSTA* Knee Surgery, Sports Traumatology, Arthroscopy; *AOTS* Archives of Orthopaedic and Trauma Surgery; *BJJ* Bone and Joint Journal; *JBJS* Journal of Bone and Joint Surgery; *J Knee Surg.* Journal of Knee Surgery

Five studies described the UCLA activity score directly before surgery, and all these studies were able to demonstrate a significant improvement after UKA implantation [10, 18, 26, 44, 45]. Only one study assessed the UCLA activity score before the onset of knee symptoms [14]. In comparison with pre-arthritic activity levels, the UCLA activity score slightly decreased from 8.1 to 7.4 points following UKA. Despite the slight decrease in activity levels, patients still had the highest UCLA activity score of all studies analysed [14] (Table 3).

Overall, nine studies used the Tegner activity score to describe the pre- and post-operative level of physical activity. Five studies reported a statistically significant improvement following UKA [21, 30, 44, 45, 47]. The remaining four studies did either show no statistical difference [18] or did not calculate a significance level, respectively [29, 33, 50]. Jahnke et al. [18] described no statistically significant difference (n.s.) in the Tegner activity score comparing pre- and post-operative levels. The mean pre-operative Tegner activity score in Jahnkes’ study was considerably higher (4.1) compared to the remaining studies using this score (Table 3). Patient demographics did not differ compared to the other studies. The reason for the high pre-operative Tegner score, therefore, cannot be explained. Schai et al. [33] reported a relative improvement from 2.3 to 2.7 points according to the Tegner activity score, without calculating a significance level. Pandit et al. [29] also described the Tegner activity score in their study of 1000 consecutive Oxford UKAs with a minimum follow-up of 10 years. There was an improvement of the pre-operative Tegner activity score up to 7 years post-operatively; however, the score decreased below pre-operative levels at 10 years. Pandit et al. [29] did not calculate any significance level. Only Yim et al. [50] described a decrease in the Tegner activity score following UKA. However, a significance level was not calculated. Yim et al. [50] described a cohort of 96 % (48 of 50) female patients (Table 3).

In a recent study on 150 consecutive cementless UKAs, Hooper et al. [15] were able to demonstrate a significant improvement according to the High Activity Arthroplasty Score following UKA (Table 3).

### Sport disciplines, length and frequency

Seven out of 17 studies described the level of physical activity in more detail, using either standardized [18, 44, 45] or non-standardized questionnaires [10, 25, 32, 50].

Using the Heidelberg Sports Activity Score, Jahnke et al. [18] reported that hiking, cycling and swimming were the three most practiced sports after surgery. Swimming, hiking and ‘other sports’ increased significantly. High-impact sports such as skiing, cross-country skiing and ball sports decreased significantly compared to pre-operatively practiced sports. Walker et al. [44] showed that biking, hiking and long walks were the most common activities after surgery according to the Schulthess Clinic Activity Score. Similar to Jahnke et al., a significant decrease in more intense activities such as tennis, soccer, jogging or skiing was observed after UKA implantation. In a further analysis of patients younger than 60 years, Walker et al. [45] reported a significant decrease in high-impact activities and a significant increase in low-impact activities after surgery, respectively.

Five studies reported physical activities based on individually developed scores. Naal et al. [25] described the physical activity using an individual score which recorded the number of activities, its frequency and the session length. The authors report an unchanged activity frequency after surgery with similar session length and a slight, although significant, decrease in the number of different sport disciplines (Table 3). The most common activities after surgery were hiking, cycling and swimming. Hopper et al. [16] analysed the participation in the following disciplines: golf, swimming, dancing (line/ballroom), bowls and cycling. The authors showed that the number of activities, its frequency and the session length remained unchanged with a UKA (Table 3). Yim et al. [50] assessed the participation in cycling, swimming, exercise walking, dancing, jogging and mountain climbing, and described a return to 1.6 activities after surgery compared to 2.2 activities before surgery, respectively. Fisher et al. [10] showed that most patients (93 %) following UKA resumed the same physical activity they performed before becoming symptomatic with knee pain. The top three activities were swimming, golf and dancing in Fishers’ study. Pietschmann et al. [32] described the percentage of patients who performed sports activities before (60 %) and after (53 %) surgery. The authors, furthermore, reported on an increase in the weekly sport frequency after UKA and a shift from high-impact sports towards low-impact sports, respectively.

### Sport participation and return to activity

The pre-operative and post-operative sport participation was described in ten studies [10, 14, 16, 18, 25, 32, 44–46, 50]. The sport participation before the onset of restricting symptoms of the knee was reported in five studies, and ranged from 64 to 93 %, respectively [10, 14, 25, 44, 45]. In all five studies, patients stayed active; however, sport participation decreased up to 9 % compared to pre-arthritic conditions (Table 4). Seven studies described the return to activity rate which ranged from 80 to 98 % [10, 14, 16, 25, 32, 44, 45] (Table 4).

**Table 4:** Summary of the reported pre- and post-operative sport participation rate and the return to activity rate of all studies analysed

Pre-operative and post-operative sport participation				
References	Time of pre-operative assessment	Pre-operative sport participation	Post-operative sport participation	Return to activity rate
Fisher et al. [10]	Before onset of knee pain	64 % (42 of 66)	59 % (39 of 66)	93 %
Walton et al. [46]	Directly before surgery	79 % (121 of 150)	86 % (131 of 131)	Not described
Naal et al. [25]	Before onset of knee pain	93 % (77 of 83)	88 % (73 of 83)	95 %
Hopper and Leach [16]	Directly before surgery	88 % (30 of 34)	85 % (29 of 34)	97 %
Yim et al. [50]	Directly before surgery	84 % (42 of 50)	60 % (30 of 50)	Not described
Pietschmann et al. [32]	Directly before surgery	60 % (78 of 131)	53 % (69 of 131)	80 %
Jahnke et al. (2014)	Directly before surgery	90 %	93 %	Not described
Walker et al. [44]	Before onset of knee pain	93 % (42 of 45)	91 % (43 of 45)	98 %
Walker et al. [45]	Before onset of knee pain	93 % (86 of 93)	91 % (85 of 93)	93 %
Ho et al. [14]	Before onset of knee pain	83 % (30 of 36)	72 % (26 of 36)	87 %

Patients with a unicompartmental knee arthroplasty participate regularly in sports

## Discussion

The most important findings of the present study were that the level of physical activity improves following UKA implantation according to established activity scores. A shift from high-impact to low-impact activities was observed. The number of different sport activities decreased, whereas the session length und frequency remained overall unchanged.

The UCLA activity score is validated activity assessment tool for patients with joint arthroplasties of the lower extremities [1, 51]. The present review demonstrates that in all but one study (*n* = 5) the UCLA activity score significantly improved following UKA implantation. The mean post-operative UCLA activity score ranged from 6.3 to 7.4 points. An UCLA activity score of 7 corresponds with the regular participation in active events such as bicycling. One study assessing the UCLA activity score before the onset of first restricting symptoms reported a slight decrease in the activity level compared to pre-arthritic conditions [14]. The study, however, still demonstrated the highest UCLA activity score of all studies analysed [14]. Overall, patients following UKA remain active according to the UCLA activity score.

The Tegner activity rating system was initially developed to evaluate activity after knee ligament injuries [38]. The Tegner and the UCLA activity scores both have a scale of 1–10 points. The Tegner score, however, is usually used for more active patient populations like ACL reconstruction patients. A Tegner score of 10, for instance, corresponds with competitive sports such as soccer on a national or international level. The mean post-operative Tegner activity score of all studies analysed in this review was 3.1 points which corresponds with competitive and recreational sports such as swimming. The fact that in some studies the Tegner score did not show statistically significant improvements following surgery is partly explained by the larger bandwidth of activities described in the Tegner activity score. The practiced activities have the change a lot to move from a Tegner activity score of 3 to a score of 4, while an improvement from level 3 to level 4 is easy on UCLA activity rating scale, respectively.

Eight out of 17 studies described the level of physical activity in more detail. All studies, irrespective of the questionnaire utilized, showed a significant decrease in high-impact activities and a significant increase in low-impact activities. The most popular activities after surgery were hiking, cycling and swimming. Almost all activities were in line with recommendations for the return to recreational and athletic activity after TKA presented by the Knee Society [12]. Naal et al. [25] reported on an active Swiss patient population of which 22 % performed downhill skiing following surgery. The Knee Society has not come to a conclusion whether downhill skiing should be recommended [13].

Although patients took up lower-impact activities, its frequency per week and the minimum session length even increased [16] or remained unchanged [25, 32]. The only study describing a decrease in activities was published by Yim et al. [50]. Interestingly, this study described an Asian cohort with 96 % (48 of 50) female patients. However, the reason for a decrease in physical activities according to the Tegner score and a sport questionnaire remains unknown.

There has been a lot of debate whether mobile-bearing designs offer a clinical advantage over fixed-bearing designs because of decreased wear rates and reduced shear stress at the interfaces [35]. However, a recent meta-analysis showed that there are no differences in the clinical outcome; and in experienced hands, revision rates are comparable [31]. The current review included four studies with fixed-bearing UKAs, and no differences in the level of post-operative physical activities were observed [25, 26, 33, 50].

Two studies reported on the level of sport and physical activity following lateral UKA [44, 47]. Both studies demonstrated a highly significant improvement of post-operative physical activity according to the UCLA and Tegner activity score, respectively.

Witjes et al. [49] recently published a literature review describing pre- and/or post-operative participation in specific types of sports and/or the time to return to sport after TKA and/or UKA. The authors concluded that participation in sports seems more likely after UKA than after TKA, and patients tend to return to lower-impact types of sport. This review is in line with these observations. However, the current review provides additional information as it included all available studies that described the level of activity utilizing scores commonly used in clinical practice.

There are a number of limitations that warrant acknowledgment. Firstly, only 8 of the 17 studies included were prospectively designed; hence, the level of evidence currently available is not particularly high. It cannot be ignored that retrospective studies may be influenced by various bias-inducing factors. Second, 12 out of 17 studies (2398 of 2636 patients) reported on the Oxford phase 3 unicompartmental knee implant. Therefore, the conclusions of this review are primarily valid for this particular type of implant. Third, most studies, with exception of Pandit et al. [29], were quite small in terms of patient numbers. Finally, the minimum follow-up was <2 years in eight studies. However, in an attempt to increase the number of studies, these studies were not excluded.

In clinical practice, this systematic review may help physicians to manage the expectations of patients regarding the level of physical activity following UKA. Eleven out of thirteen studies described a post-operative increase in physical activity according to validated activity scores. Almost all activities were in line with Knee Society recommendations for the return to recreational and athletic activity. Even though patients have increasing demands, this report suggests that patients do not participate in high-level sports following UKA. The reason for a limitation of sport activities remained unclear in most studies. Only Walker et al. [44] described that the preservation of the implant was the main reason for a limitation of activities.

Additionally, this study allows surgeons performing UKAs to compare their results with the results published in the literature.

## Conclusion

Patients following UKA are active according to validated activity scores. A significant increase in low-impact activities and a decrease in high-impact activities after UKA was observed. The number of different sport activities decreased, whereas the session length und frequency remained overall unchanged. Patients with a UKA regularly participate in sport, however, sport participation slightly decreased compared to pre-arthritic levels.

## Appendix

The following search terms were used on MEDLINE:

1. UKA.
2. (activity, physical[MeSH Terms] OR activity, motor[MeSH Terms] OR sports[MeSH Terms] OR sports OR sport OR (motor AND activity) OR motor activity OR physical activity OR activity).
3. 1. AND 2.
